# Zinc Oxide Nanoparticles and Their Biosynthesis: Overview

**DOI:** 10.3390/life12040594

**Published:** 2022-04-18

**Authors:** Hareb Al Jabri, Muhammad Hamzah Saleem, Muhammad Rizwan, Iqbal Hussain, Kamal Usman, Mohammed Alsafran

**Affiliations:** 1 Center for Sustainable Development (CSD), College of Arts and Sciences, Qatar University, Doha 2713, Qatar; h.aljabri@qu.edu.qa; 2 Department of Biological and Environmental Sciences, College of Arts and Sciences, Qatar University, Doha 2713, Qatar; 3 Office of Academic Research, Office of VP for Research & Graduate Studies, Qatar University, Doha 2713, Qatar; saleemhamza312@webmail.hzau.edu.cn (M.H.S.); m.rizwan@qu.edu.qa (M.R.); 4 Department of Botany, Government College University, Faisalabad 38000, Pakistan; driqbal@gcuf.edu.pk; 5 Agricultural Research Station, Office of VP for Research & Graduate Studies, Qatar University, Doha 2713, Qatar; 6 Central Laboratories Unit (CLU), Office of VP for Research & Graduate Studies, Qatar University, Doha 2713, Qatar

**Keywords:** nanoparticles, plant growth, elements, artificial chemicals, agricultural system

## Abstract

Zinc (Zn) is plant micronutrient, which is involved in many physiological functions, and an inadequate supply will reduce crop yields. Its deficiency is the widest spread micronutrient deficiency problem; almost all crops and calcareous, sandy soils, as well as peat soils and soils with high phosphorus and silicon content are expected to be deficient. In addition, Zn is essential for growth in animals, human beings, and plants; it is vital to crop nutrition as it is required in various enzymatic reactions, metabolic processes, and oxidation reduction reactions. Finally, there is a lot of attention on the Zn nanoparticles (NPs) due to our understanding of different forms of Zn, as well as its uptake and integration in the plants, which could be the primary step toward the larger use of NPs of Zn in agriculture. Nanotechnology application in agriculture has been increasing over recent years and constitutes a valuable tool in reaching the goal of sustainable food production worldwide. A wide array of nanomaterials has been used to develop strategies of delivery of bioactive compounds aimed at boosting the production and protection of crops. ZnO-NPs, a multifunctional material with distinct properties and their doped counterparts, were widely being studied in different fields of science. However, its application in environmental waste treatment and many other managements, such as remediation, is starting to gain attention due to its low cost and high productivity. Nano-agrochemicals are a combination of nanotechnology with agrochemicals that have resulted in nano-fertilizers, nano-herbicides, nano-fungicides, nano-pesticides, and nano-insecticides being developed. They have anti-bacterial, anti-fungal, anti-inflammatory, antioxidant, and optical capabilities. Green approaches using plants, fungi, bacteria, and algae have been implemented due to the high rate of harmful chemicals and severe situations used in the manufacturing of the NPs. This review summarizes the data on Zn interaction with plants and contributes towards the knowledge of Zn NPs and its impact on plants.

## 1. Introduction

### 1.1. Zinc

Zinc (Zn) is one of the primary micronutrients involved in plant growth and production. It also has a constituent that is required in small amounts for several enzymes and protein activities [1,2,3]. Most enzymes, including carbonic anhydrase, carboxypeptidase, and superoxide dismutase, require Zn as a cofactor [4,5]. Zn deficiencies can affect a plant by stunting its growth, decreasing the number of tillers, causing chlorosis and smaller leaves, increasing crop maturity period, and causing spikelet sterility and inferior quality of harvested products [6,7]. Plant enzymes activated by Zn are involved in carbohydrate metabolism, maintenance of the integrity of cellular membranes, protein synthesis, regulation of auxin synthesis, and pollen formation [8,9]. The regulation and maintenance of the gene expression required for the tolerance of environmental stresses in plants are Zn dependent [10]. Zinc seems to affect the capacity for water uptake and transport in plants and also reduce the adverse effects of short periods of heat and salt stress [5,11]. As Zn is required for the synthesis of tryptophan, which is a precursor of IAA, it also has an active role in the production of an essential growth hormone auxin [12]. Zn accumulation in soils is of great concern in agricultural production due to its adverse effects on food safety and marketability, crop growth due to phytotoxicity, and the environmental health of soil organisms [13]. In addition, Zn contamination in the soil may pose risks and hazards to humans and the ecosystem through direct ingestion or contact with contaminated soil, the food chain (soil-plant-human or soil-plant-animal human), drinking of contaminated groundwater, reduction in food quality (safety and marketability) via phytotoxicity, reduction in land usability for agricultural production causing food insecurity, and land tenure problems [4,14].

Zn contamination issues are becoming increasingly prevalent, with many documented cases of metal toxicity in mining industries, foundries, smelters, coal-burning power plants, and agriculture [15]. The average range of Zn required by the plant is 15–55 ppm and in the growing medium between 0.10 to 2.0 ppm. Zinc toxicity and deficiency have an adverse effect on the yield and crop damage [16,17]. Organic matter plays a significant role in maintaining the availability of Zn in soil. It promotes Zn uptake by roots by releasing Zn over time and changing the physicochemical features of the soil, which increases Zn availability in the soil [18]. Extractable Zn increases with the increases in the organic carbon content in the soil [19], and Zn solubility in soils is improved by adding organic matter [20].

### 1.2. Plant Absorption of Zn

Zn is essential for the growth in animals, human beings, and plants, and is vital to the crop nutrition as it is required in various enzymatic reactions, metabolic processes, and oxidation reduction reactions [21,22]. In addition, Zn is also essential for many enzymes which are required for nitrogen metabolism, energy transfer, and protein synthesis [23]. Depending on the nature of experiments and plant species, the most significant mechanisms may be Zn utilization in tissues and Zn uptake [9,24]. Under Zn deficient conditions, Zn-efficient genotypes have a high activity of Cu/Zn anhydrase and carbonic anhydrase. Zn efficiency and Zn uptake are very important for plant growth and its total content in soil is influenced by several soil properties like pH, CaCO_3_, organic matter content, and type of crop, as well as cultivars and nutrient interactions in soil environment [19,25].

**Figure 1:**
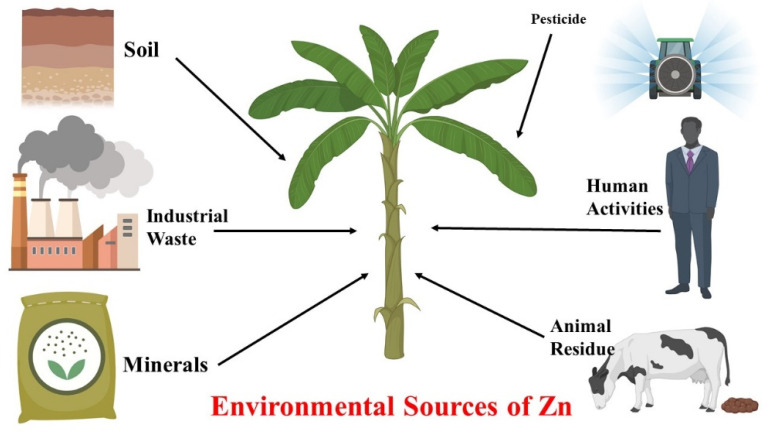
Sources of Zn (nutritional form for plants) from different environment sources.

Zn is mostly absorbed by roots from the different homogenous content in the soil, in the form of Zn^2+^ ions or in the form of organic acid chelates [4], and translocated into the above-ground section of the plant via the xylem [26]. It was also reported that the Zn can also be absorbed by plants through their leaves through various applications such as foliar spray [27]. However, the mechanism behind it is yet unknown. The surface characteristics of the leaves influence the transport of nutrients; this has been reported for many other nutrients, such as Cu [28,29] and Fe [30,31]. The thickness of the waxy covering on the leaf and the chemical composition of the cuticle, as well as its density, trichomes, and stomata, are all factors to be considered for the absorption of Zn through the leaf area [12,32]. The absorption of Zn through the various sources of the environment under different conditions are presented in Figure 1.

### 1.3. Effect of Zn on Plant Growth

Zn is required for the activation of many enzymes in plant cells, such as alcohol dehydrogenase, carbonic anhydrase, and RNA polymerase [3,5]. Zn is also involved in biomembranes’ stabilization by interacting with the phospholipids and sulfhydryl groups of membrane proteins [33,34]. It can contribute to proteosynthesis, metabolism of carbohydrates, and lipid and nucleic acid synthesis. Furthermore, Zn plays a crucial role in oxygen radical production, as well as their detoxification [24]. Zn participates in Cu-Zn-SOD enzyme synthesis, a key enzyme involved in the removal of toxic O^−^_2_ radicals, which can be harmful to membrane lipids and proteins [21,35]. Cu-Zn-SOD is essentially localized in chloroplasts; in some plants, it is found in the thylakoid lumen whereas in others it is bound to the thylakoid [36,37]. Deficiency of the Zn is the common micronutrient deficiency concern, affecting practically in all crops [34]. Zn deficiency can be found in every part of the world and almost all crops respond positively to application of Zn [38] and can cause a plant’s growth to be stunted, resulting in fewer tillers, lower rate of chlorosis, and smaller leaves, as well as a longer crop maturation period, and lower quality harvested crop [39]. Normal soils inherit their trace elements, which include Zn primarily from the rocks through geochemical and pedochemical weathering processes [23]. Besides mineralogical composition of the parent material, the total amount of Zn present in the soil is dependent on the type of soil, intensity of weathering, climate, and numerous other predominating factors during the process of formation of Zn in the soil in the form of sulphate or oxide, enhance overall shoot growth. Shoot growth was 21.6 percent higher in Zn-treated plants than in control plants, when chickpea was foliar sprayed by Zn-O nanoparticles; however, there was evidence of a negative influence on root growth, the shoot to root ratio was somewhat altered as a result [40]. This is in contrast with the findings of Prasad et al. [41] in peanut, using 400–2000 ppm nano Zn-O, which showed an improved response in terms of the shoot and root growth. Zn application has also been shown to change the root: shoot ratio in various genotypes of rice [42], spinach [43], and wheat genotypes [6].

### 1.4. Protective Role of Zn in Plants

Zn is a fundamental nutrient for plants as it plays a vital role as metal component and co-factor of many enzymes [10]. The cell membrane is the first target of abiotic stresses [44,45], and the maintenance of its stability under harsh environment is the core part of plant tolerance [43,46]. Adequate Zn supply in a stressed environment maintains membrane permeability, the activity of antioxidant substances, photosynthetic efficiency, and water use efficiency [37,47]. Moreover, Zn application results in an appreciable increase in leaf area, the content of chlorophyll, and other photosynthetic pigments and stomatal conductance, thus resulting in improved growth and yield [10,18]. In addition, Zn is a catalytic and structural protein cofactor found in many of enzymes, and it plays an important structural part in protein domains [48]. The “Zn finger” proteins are involved in transcription factor DNA binding as well as protein–protein interactions [49]. Bioinformatic techniques can now predict Zn-binding sites using sequenced metal-binding motifs [7].

**Figure 2:**
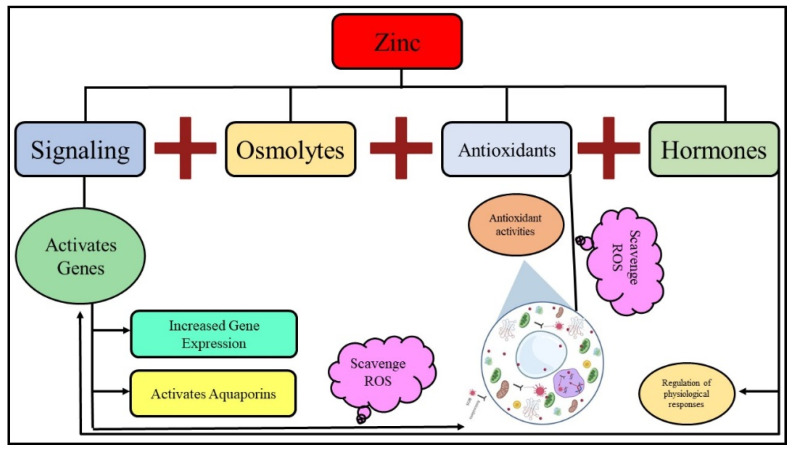
Mechanism of abiotic stress tolerance induced by the application of Zn. Zn application improves the antioxidant activities, increases osmolyte accumulation, hormonal cross talk and cell signaling, which, in turn, improve membrane stability and physiological processes, including water uptake and ROS scavenging.

Zn plays a pivotal function in the plant response to pests and diseases. Nonetheless, Zn defense-related mechanisms in plants greatly vary. The outcomes of plant–pest/pathogen interactions differ, depending on the effectivity of the Zn-related responses in limiting the invader’s attack as well as on the enemy’s ability to circumvent the plant defenses, in addition to other environmental conditions that can favor either host or invader [50,51]. Several studies have shown that, in most cases, Zn fertilization decreased plant symptoms [52]. However, a protective Zn concentration against certain pathogens can also induce a higher susceptibility to another pathogen on the same plant [53]. Several studies have demonstrated that the Zn application decreases plant diseases/symptoms in the majority of cases [3,21,49]. Therefore, Zn application counteracts environmental stress by improving membrane stability, hormone synthesis, the photo-synthetic process, and the scavenging of reactive oxygen species (ROS) (Figure 2).

### 1.5. Proteins with Zn Fingers

In addition to their role in plant growth and development, Zn finger proteins regulate plant responses to biotic stress conditions [54]. Zn finger protein possesses one or more ‘Zn finger’ that bond one or more Zn ions by its residues Histidine and Cysteine. The Zn finger protein also belongs to a large family of transcription factors. It plays many important regulatory roles in plants [55]. The Zn finger domain enables different proteins to interact with or bind DNA, RNA, or other proteins, and is present in the proteomes of many different organisms. Proteins containing Zn finger domain(s) were found to play important roles in eukaryotic cells regulating different signal transduction pathways and controlling processes, such as development and programmed cell death [56]. There are many types of zinc finger proteins, classified according to the number and order of the Cys and His residues that bind the Zinc ion [57]. With a broad spectrum of structures and functions, these proteins are defined as those with a small, freely folded functional domain that requires one or more Zn ions to stabilize its structure. Zn finger binding domains are present in the well-known plant resistance proteins NBS-LRRs (nucleotide binding sites-leucine rich) that are involved in the effector-triggered immune response [58]. The authors analyzed seventy plant disease resistance proteins from various crops. Zn finger domains are found in 37% of these proteins, implying that this protein family plays a significant part in the host’s resistance to infections [32,58]. RAR1 (Zn-binding protein in wheat) also provides resistance to rust pathogen via an oxidative burst and hypersensitive response mediated by salicylic acid [55]. The up-regulation of the 2 zinc-finger transcription factors in potatoes has been linked to insect invasion [54].

## 2. Role of Zn in Plant Nutrition

Zn is a key plant nutrient, ranked the third most dominant metal after Fe and Mn. Zn mediates a number of plant metabolic/biochemical/physiological reactions [1,13]. Biofortification refers to improvement in food nutritional quality via different techniques such as agronomic activities, recombinant DNA technology or conventional plant breeding. Zn influences plant metabolism by regulating the activities of hydrogenase and the carbonic anhydrase, and the stabilization of ribosomal fractions and cytochrome synthesis. Enzymes that are triggered by the Zn are involved in the metabolism of glucose, cellular membrane integrity, synthesis of protein, auxin production control, and pollen development [59]. Zn deficiency in plants may provoke several symptoms, such as chlorosis, necrosis, spikelet sterility, enhanced membrane permeability, stunted growth, leaf bronzing, small leaves, thin stem, and even shoot dieback [10,60,61]. Zn deficiency symptoms generally appear 2–3 weeks after exposure to Zn deficient conditions. Zn-deficiency-mediated visual symptoms only appear under severe conditions. While the marginal Zn deficiency only affects plant yield without the visual symptoms [26]. The induction of oxidative stress under Zn deficiency is another well-known mechanism at the cellular level [62]. Several reports revealed that low levels of Zn in plants mediate enhanced levels of ROS that may be due to the lower concentration of Cu-Zn-SOD enzyme [63]. Although Zn storage in cell vacuoles is a tolerance strategy against Zn toxicity, its remobilization is also important during deficient conditions [59,64]. Moreover, Zn reserves in the vacuoles are remobilized when required in other parts of the plant. Members of the NRAMP family might help the efflux of metals from vacuoles [32].

### 2.1. Interactions of Zn with Other Nutrients

Zn is now an integral part of fertilizer recommendation for most crops in several countries. It is generally applied along with NPK as basal fertilizer at seeding (transplanting in case of rice) although its foliar application is also recommended [20]. Soil application has the advantage of leaving residual effects on succeeding crop and, thus, permitting a better utilization of applied Zn in a cropping system [65,66]. The interaction of Zn with other plant nutrients in soils and plants has aroused considerable interest in the researchers, students, planners, and academics. An interaction between two nutrients is considered statistically significant when the level of application of one nutrient affects the response of plants to the other nutrient and vice versa. When the response of plants to one nutrient increases with an increase in the level of the other nutrient, the interaction is said to be positive, and the nutrients are said to be synergistic. On the other hand, when the response to one nutrient decreases with an increase in the level of the other nutrient, the interaction is said to be negative, and the two nutrients are said to be antagonistic. In plants, Zn interacts positively with N and K and negatively with P, Ca, Fe, and Cu [36,55]. The negative interaction is due to interference of P, Ca, Fe, and Cu in the absorption of Zn on root surfaces or/and its translocation from root to shoot in plants [40]. Zn interacts negatively with Ca mainly because it competes for the same adsorption sites on soil particles as well as on root surfaces [67]. Regarding S, both positive and negative interaction effects are reported in crop plants, suggesting different mechanisms in different plant species [68]. Zn interferes with the absorption of Fe and B by plants [69,70]. Application of Zn is suggested as a measure to alleviate B toxicity in crops grown on boron-rich soils [52]. On the other hand, Zn fertilization augments the absorption of Cu and manganese by plants [71].

### 2.2. Interactions between P and Zn

Increased sorption of Zn in soils, due to increased negative surface charges associated with applied P reducing its availability, has been reported [72,73]. Zn can also be precipitated as Zn phosphate with the addition of phosphate fertilizers. The study of the interaction of P and Zn began in 1936; this was the fundamental plant growth problem which is still being discussed today. P-induced Zn deficiency is the common name for this interaction. This type of plant growth issue is linked to high quantities of accessible P or the administration of P to the soil. The mechanism and processes are still unknown. It was observed that prior heavy P application in five Hawaiian soils had no influence on DTPA extractable Zn and concluded that Zn deficiency could not be due to precipitation of Zn as insoluble ZnP compounds [74]. The production of the insoluble Zn_3_ (PO_4_)_2_ in soil was thought to have lowered Zn content in the soil to inadequate levels [75].

### 2.3. N-Zn Interactions

With the administration of N fertilizers, Zn deficiency in plants can be alleviated. The application of N increases plant development and to a smaller extent, changes the pH of root surroundings, therefore, beneficial interactions between rising levels of the Zn and N fertilizers have been found [76]. Shivay et al. [77] showed that N concentration in chickpea (*Cicer arietinum*) increased from 36.1 mg kg^−1^ in check (no-Zn) to 47.2 mg kg^−1^ with an application of 7.5 kg Zn ha^−1^. They also reported that increase in N concentration in chickpea grain was significantly greater with foliar application of Zn than with soil application, and for foliar application, Zn-EDTA was a better source than Zn sulphate. In the absence of NH_4_NO_3_ fertilizer, wheat grown on N-deficient soil with appropriate levels of all the nutrients except N and Zn did not respond to Zn treatment [78]. Moreover, Kutman et al. [78] suggested that N increased Zn uptake by roots as well as its translocation to the shoot. However, high levels of N leading to excessive vegetative growth rate may induce Zn deficiency in plants on Zn-deficient soils N fertilizers, on the other hand, have improved (or aggravated) Zn deficiency in soils that are less in Zn but high infertility by influencing Zn absorption through pH changes [79].

### 2.4. Interaction between Macronutrients

**Figure 3:**
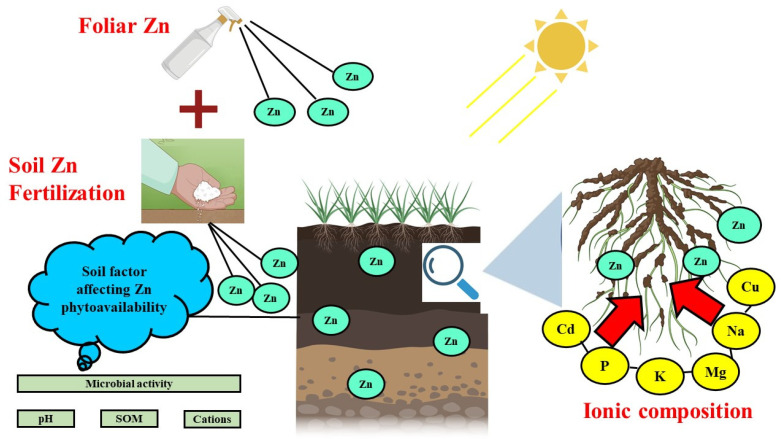
Interaction of Zn with other micronutrients in the soil.

Antagonistic effects of Calcium (Ca), magnesium (Mg), potassium (K), and Zn have been known since long. In addition, Ca, Mg, and K, among other macronutrient cations, prevent plants from absorbing Zn from the medium. They must be taken into account when interpreting the findings of Zn nutrition solution culture tests; nevertheless, in the soil, it appeared less efficient in inhibiting the absorption of Zn than the effects on pH of soil. The highest concentrations of Zn are found in the legumes [80]. It was also observed that the increasing Ca (NO_3_)_2_ concentrations from the range 0 to 40 mM decreased Zn absorption rate by the wheat seedlings, but that high concentrations of Ca (100 mM) had no effect on the absorption of Zn [81]. Ca was considered for the inhibition since changing the anions had minimal influence on the absorption of Zn, although replacing other cations for the Ca have a similar negative effect. In addition, Ca plays an important role in cell permeability and stabilization of plasma membrane by Ca under Zn toxicity conditions has been reported [50]. Application of 47.4 kg K ha^−1^ combined with foliar application of 57.6 g Zn ha^−1^ and 1728 g P ha^−1^ improved yield of Egyptian cotton (*Gossypium barbadense* L.) [82]. Marschner et al. [83] reported that in Zn-deficient soils application of Mg increased Zn concentration in beans (*Phaseolus vulgaris*) and application of Zn increased Mg concentration. Thus, a positive interaction exists between Zn and Mg. The interactions between Zn and other nutrients in the agricultural soil is presented in Figure 3.

## 3. Role of Zn in Metal-Contaminated Soil

Metal contamination issues are becoming increasingly common in all over the world, with many documented cases of metal toxicity in mining industries, foundries, smelters, coal-burning power plants, and agriculture [84,85,86]. Heavy metal accumulation in soils is of great concern in agricultural production due to its adverse effects on food safety and marketability, crop growth due to phytotoxicity, and the environmental health of soil organisms [87,88,89,90]. In addition, heavy metal contamination of soil may pose risks and hazards to humans and the ecosystem through direct ingestion or contact with contaminated soil, the food chain (soil–plant–human or soil–plant–animal human), drinking of contaminated groundwater, reduction in food quality (safety and marketability) via phytotoxicity, reduction in land usability for agricultural production causing food insecurity, and land tenure problems [91,92,93,94,95]. Zn plays a vital role in proteins, nucleic acids, and auxin synthesis, as well as antioxidation and detoxification, and are considered essential mineral elements for the plants under metal-stressed conditions [96]. Zn is a nutrient element in plant growth and antagonizes the absorption of many heavy metals, due to similar chemical properties of the many trace metals [36]. Previous studies have shown that foliar application with Zn could effectively reduce the metal concentrations in the plants. For instance, Sarwar et al. [97] found that foliar application of ZnSO_4_ at a concentration of 0.3% could effectively prevent the adverse impacts of Cd exposure and reduce the wheat grain Cd concentrations by more than 18% for plants grown in Cd-contaminated soils.

Zn was found to not only reduce the harmful levels of various metals, but also improve the plant development characteristics by blocking heavy metal uptake by plant sections [12]. However, the concentration of metal in the plant exceeds a critical value; toxicity symptoms appear, including reduced yield, poor seed germination, stunted leaf, and root growth; and ultrastructural and anatomical alterations occur, leading to the formation of reactive oxygen species (ROS) [93,98,99,100,101]. A direct effect of excess metal in the soil is the lipid peroxidation of cellular organelles that promotes ROS accumulation and impairs the functioning of the cell membrane system [30,102,103,104]. Zn reduces heavy metal toxicity in plants by developing antioxidant defenses against oxidative damage and improve plant growth and development by reducing metal toxicity and metal concentration in the body parts of the plants [96]. The effect of Zn application in various forms under the different plant species, when grown in the metal-contaminated soil, is presented in Table 1.

## 4. Nanoparticles

The term “Nano” is derived from the Greek word ‘Nanus’, which means “dwarf.” When a meter is divided into 100 billion parts (10^−9^), we have reached at a new scale known as nanoscale [111,112]. Nanotechnology is a technique that uses the nanoscale in at least one dimension and has applications in a variety of fields, which include medicine, agriculture, food, and pharmaceuticals [36,113]. Nanoparticles (NPs) are essential due to their physical, chemical, and magnetic properties, and the fact that they are inexpensive, safe, and environmentally friendly [21,62,114]. Although “dimension” is one of the fundamental features of NPs, some NPs, such as quantum dots and carbon dots, have no dimensions (metal NPS) [107]. Nanotechnology is a rapidly developing technology that has the potential to revolutionize every aspect of research [115]. This technology is employed in optics, electronics, medicinal, and materials sciences, among other fields [116]. Nanotechnology is concerned with nanoparticles, which are aggregates; their size is approximately 100 nanometers. These nanoparticles are altered forms of the basic elements that are created by changing their atomic characteristics [36,117]. Due to their strange and interesting features, nanoparticles have received a lot of attention. Nanotechnology is a popular topic in modern scientific study. This technology has a wide range of novel applications, including food processing and agricultural production, as well as advanced medicinal approaches (Sahoo 2010). The production, characterization, and study of materials in the nanoscale range (1–100 nm) is referred to as nanotechnology. The features of the living and manmade systems are studied at this level [118].The structure of these particles, due to their size, significantly increased chemical and biological properties. Nanoparticles (NPs) have larger surface areas than macro-sized particles due to their nanoscale size [51,62,119].

At the atomic level of (1–100 nm), NPs are known as modified particles. They have size-related characteristics that differ greatly from bulk materials [110,120]. Metal NPs’ intrinsic features, such as Zn oxide, titanium dioxide, and silver, are primarily defined by their size and shape. The chemical, mechanical, electrical, structural, and optical properties of materials can be altered by shrinking them to the nanoscale. These changed properties permit NPs to interact with cell biomolecules in a unique way, making the physical transport of NPS into inner cellular structures easier [51,114].

### 4.1. Methods for Synthesis of Nanoparticles

For the synthesis of the NPs, two methods are proposed: a bottom-up and a top-down approach are employed. In the top-down technique, the grinding of big macroscopic particles is conducted. It entails first creating large particles, then shrinking them down to the nanoscale via plastic deformation [121]. It is an expensive and time-consuming process; this approach cannot be used for large-scale nanoparticle production. The most popular technique for nanomaterial creation that uses a top-down approach is interferometry lithography [122]. The nanoparticles show enhanced properties, such as high reactivity, strength, surface area, sensitivity, stability, etc., due to their small size. The nanoparticles are synthesized by various methods for research and commercial uses that are classified into three main types, namely physical, chemical, and mechanical processes that have seen vast improvements over time.

### 4.2. Zn-NPs

There are several types of Zn nanoparticles, such as ZnS and ZnSe, or quantum dots CdSe/ZnS. Many of them can be modified to have more or better fluorescent properties, which is why they are under consideration for future use in protein determination, immunofluorescence analysis, immunohistochemical detection, and 3D confocal study of membrane proteins [34,119]. Probably the most widespread type of zinc nanoparticles (in practice as well as research) is Zn oxide (nano-ZnO). The normal ZnO and its nanoparticles are commonly added to plastic, glass, ceramics, cement, and rubber materials, as well as pigments, paints, food supplements, batteries, and non-flammable materials. The reason for this is their wide range of suitable properties, which is also linked with the easy availability and low price of the chemical. These properties include relatively high electrical and thermal conductivity and stability in high temperatures with a neutral pH and mild antimicrobial effects [34]. ZnO nanoparticles are, thanks to their photostability and ability to absorb UV radiation, also used in cosmetic products and sunscreens. An estimated 10,000 tons of UV filters are produced annually for the world market, and there are approximately 550 tonnes of ZnO nanoparticles alone being produced worldwide [109,119].

### 4.3. NPs of ZnO

**Figure 4:**
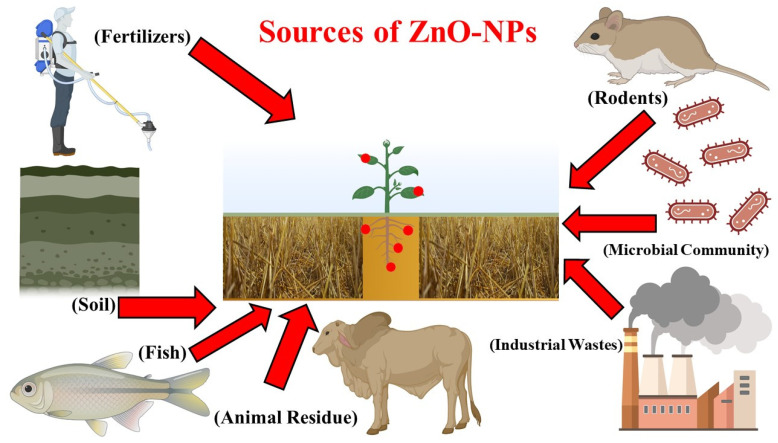
ZnO-NPs in soil and their uptake.

ZnO is the chemical formula of ZnO and is an inorganic substance. It is in the form of a white-colored powder of that is insoluble in water [110]. Paints, adhesives, plastics, sealants, pigments, food, ointments, batteries, ferrites, and fire retardants are just a few of the materials and products that use ZnO powder as an addition. It is found in Earth crust in the form of zincite mineral, although most ZnO utilized for commercial applications is synthesized [49]. Zn and oxygen correspond to the second and sixth groups in the periodic table. ZnO is commonly referred to as an II-VI semi-conductor in materials science. ZnO is a flexible, useful, and a strategic inorganic substance with a wide range of uses. It is called an II-VI semiconductor [123] because Zn and O belong to the periodic table’s groups two and six, respectively. The optical characteristics of ZnO are all unique [124]. It has a large bandgap of (3.3 eV) in the ultraviolet spectrum, at room temperature has high binding energy, and high electrical conductivity that is of n-type [26,36]. Different sources of ZnO-NPs are presented in Figure 4.

Plastic and rubber products typically contain ZnO and its nanoparticles. This is due to its extensive range of acceptable qualities, which is linked to the chemicals that are easily available at low prices [62]. The ZnO nanoparticle’s benefits were demonstrated multiple times; nevertheless, the high concentration cannot be used. The spraying of ZnO nanoparticles size of 25 nm at a dosage of 1000 mg/L on peanuts (*Arachis hypogaea*) resulted in a considerable increase in germination. The plant bloomed earlier, and the chlorophyll content increased [41].

### 4.4. Plants and Modified ZnO-NPs

In the plant world, ZnO is not the only sort of nanoparticle being studied. Experiments with coat changes on Zn-NPs have also been conducted. Yuvaraj et al. [125] developed manganese-coated ZnSO_4_ nanoparticles. Mukherjee et al. [126] investigated the toxic effects of ZnO-NPs on pea plants (*Pisum sativum* L.). He explained that when the ZnO nanoparticles were covered with iron, it reduced the toxic effect; the modified ones had no deleterious impact on germination and did not significantly reduce chlorophyll concentration [127].

### 4.5. ZnO Nanostructures Synthesis

Due to its multifunctional qualities in a variety of applications, ZnO nanostructures have been the subject of extensive research. Nanostructures of ZnO have arisen as a promising candidate for energy harvesting, and a wide range of electrical devices. Several notable uses are now being investigated in the field of biomedical and in the anti-viral field. This is due to their possible biocompatibility in comparison to other metal oxides, alkaline solubility, and Zn-O terminated polar surfaces [26].

## 5. ZnO-NPs Synthesis by Chemical Methods

Some of the common processes that are used to create nanomaterials or nanostructures are explained in the table (Table 2).

### 5.1. Benefits of Chemical Methods

It is a significant process, and it may be conducted with a variety of precursors and under a variety of variables like temperature, time, the concentration of reactant, and so on. The size and geometries of the resultant nanoparticles are morphologically different when these parameters are changed. The various chemical processes for producing ZnO-NPs are given below.

### 5.2. Reaction of Zn and Alcohol

Alcohol is used for the synthesis of ZnO by chemical methods. Some amount of ethanol is mixed with zinc powder. This mixture is heated at a high temperature for some minutes, then the solution is kept at room temperature for two days. The product is extracted from the resultant suspension, centrifuged, washed, and vacuum dried. Oxide particle development is sluggish and controlled in alcoholic medium [133].

### 5.3. Vapor Transport Synthesis

The vapor transport approach is the most prevalent method. ZnO nanostructures are formed when Zn and oxygen react. ZnO vapor can be produced by a variety of methods. Another direct way is to heat zinc powder in the presence of oxygen, although the growth temperature is relatively moderate. The ratio of Zn vapor pressure and oxygen pressure must be carefully managed to acquire appropriate ZnO nanoparticles [129].

### 5.4. Hydrothermal Methodology

Due to low process temperatures, this technique is an effective method for controlling particle size. This approach offers various advantages over the growth procedures, including the use of simple apparatus, catalyst-free growth, less expensive, and homogeneous production, as well as being eco-friendly and less toxic. Due to the low reaction temperatures, this approach is appealing to microelectronics. This method has been used to make ZnO NPs and other luminous materials with great success [130].

### 5.5. ZnO-NPs Green Synthesis

**Figure 5:**
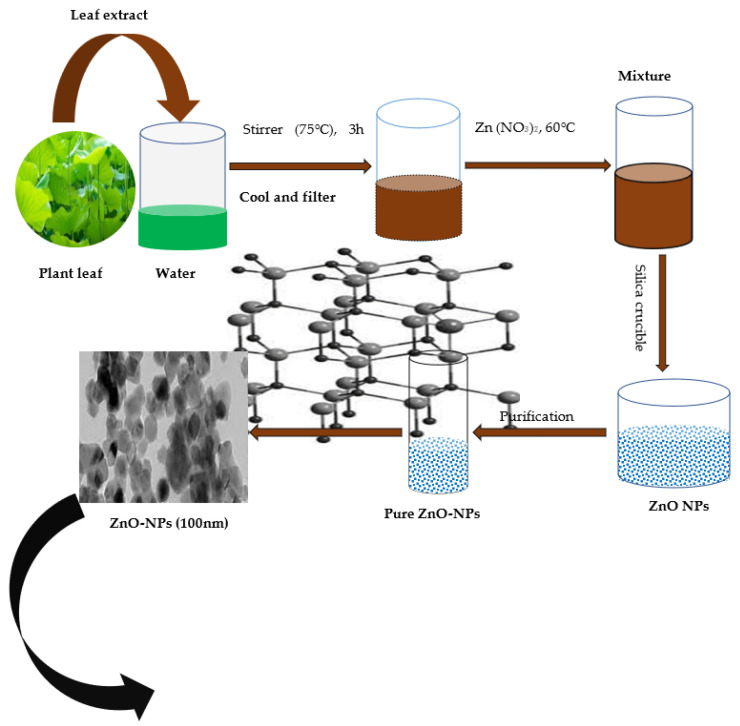
Green Synthesis of ZnO-NPs.

Owing to the growing popularity of green methods, several methods have been implemented to produce ZnO-NPs using different sources, such as bacteria, fungus, algae, plants, and others. A list of tables was prepared to summarize the research carried out in this field [109]. The synthesis of biological nanoparticles represents an alternative for the physical and chemical methods of nanoparticle formation. The majority of researchers focused on the green synthesis of nanoparticles for the formation of metal and oxide nanoparticles. The use of plants for the synthesis of nanoparticles is a rapid, low-cost, eco-friendly option and is safe for human use [34]. *Vitex negundo* plant extract was used to produce ZnO NPs with zinc nitrate hexahydrate as a precursor. The biosynthesized ZnO NPs showed antimicrobial activities against *E. coli* and *S. aureus* bacteria [134]. Several biological systems are utilized safely in biogenic NPs synthesis. However, employing microorganisms to make nanoparticles is difficult due to the lengthy process of maintaining the cell cultures, intracellular production, and many purifying stages. Due to the unique phytochemicals that they produce, plant components are employed to make ZnO NPs. Extracts of plant parts are an eco-friendly, less expensive method that does not require the use of middle base groups (Figure 5). It takes a fraction of the time, requires no expensive equipment or precursors, and produces highly quantity products devoid of contaminants [21].

The most popular source of NP synthesis is plants because they allow for large production as well as the generation of stable NPs with a variety of sizes and shapes [135]. Phytochemicals released by the plant are used to reduce metal ions 0 valences [30]. The most popular approach for preparing ZnO-NPs from plant parts is to thoroughly wash the plant component in running water and sanitize it with distilled water. The plant component is then allowed to dry at room temperature before being weighed and crushed. Milli-Q water is then added to the plant portion, and the mixture is cooked with constant stirring. The solution is filtered using filter paper and the remaining solution is used as the plant extract. To accomplish efficient mixing, a portion of this extract is mixed with an amount of Zn nitrate, ZnO, and the combination is heated at the proper temperature. At this point, some people experiment with temperature, pH, extract concentration, and duration to see what works best. The mixture turns yellow after the incubation period, which is visible evidence of the produced NPs [121].

ZnO-NPs can be synthesized through many physiochemical routes, such as sol-gel processes, co-precipitation, laser vaporization, microemulsion, and ball milling [30]. Commonly, these preparation methods face several limitations, such as the high cost of equipment, the large area required for equipment set up, and additional use of capping agents, stabilizers and toxic chemicals [134]. Most of these chemical methods are not environmentally friendly due to the use of harsh chemicals for stabilizing processes, which will bind to the ZnO-NPs and limit their biological applications [109]. To overcome these limitations, green chemistry procedures have attracted significant scientific attention and have provided a new path for material researchers because they are safe and environmentally friendly methods, which do not produce toxic by-products [130]. Developing simple and green methods for synthesizing ZnO-NPs is, thus, important, and remains a challenge for researchers [133]. Biosynthesis of NPs refers to the synthesis of NPs using plants or microorganisms. NPs from such “green synthesis” have been used in the field of drug, gene delivery, and various medical treatments including antimicrobial, anticancer, anti-inflammatory, antiaging, antioxidant, and anti-biofilm inhibition [132]. Oxide NPs synthesized using eukaryotic organisms such as fungi are beneficial due to their ability to produce a large amount of enzymes [132]. In addition, there are three procedures which have been frequently chosen for the preparation of ZnO-NPs. These procedures are categorized as {ZnAc (zinc acetate dihydrate), 2-Methoxyethanol, MEA}, {ZnAc, 2-Propanol, DEA}, and {ZnAc, Ethylene Glycol, glycerol, 1-Propanol,}. In some studies, a simple, green, cost-effective ultrasound assisted coating of ZnO-NPs on the paper surface, without the aid of binders, have been reported. The paper surfaces coated with ZnO-NPs are characterized using scanning electron microscope (SEM), X-ray diffraction (XRD), and attenuated total reflectance-Fourier transform infrared (ATR-FTIR). Loading of ZnO-NPs on the paper surface is estimated from the thermogravimetric analysis (TGA). Furthermore, time-of-flight secondary ion mass spectrometry (TOF-SIMS) has been used to characterize the surface composition of the coated surface, binding sites of the NPs, and distribution of the coated ZnO-NPs [131].

### 5.6. Bacterial-Based Green Synthesis of ZnO-NPs

Although this technique is green-based, it shows some drawbacks, including time-consuming microbe screening; it is also time-consuming and expensive. *B. licheniformis* generated ZnO nanoflowers using a green synthesis method that showed photocatalytic activity. The nanoflowers displayed increased photocatalytic activity and it is thought that the bigger oxygen vacancy in the produced NPs is what gives them this ability. Photo-catalysis produces active species by absorption of light [132].

### 5.7. Microalgae and Macroalgae Are Used in the Green Method of ZnO-NPs

Algae are photosynthetic organisms that can be unicellular (like Chlorella) or multicellular. Algae are devoid of basic plant structures such as roots and leaves. Rhodophyta, Phaeophyta, and chlorophytes are the three types of marine algae. Algae have been extensively used in the production of nanoparticles of Au and Ag, but their use in the synthesis of ZnO-NPs is narrow and documented in a few works [136].

### 5.8. Benefits of Green Synthesis of NPs

The idea emphasizes the use of environmentally friendly reagents. Although physical and chemical approaches for nanoparticle manufacturing are faster and easier, the biogenic process is more effective and environmentally friendly. It also decreases pollution [137].

### 5.9. Nano Agrochemicals

Nano-agrochemicals are a combination of nanotechnology with agrochemicals that have resulted in nano-fertilizers, nano-herbicides, nano-fungicides, nano-pesticides, and nano-insecticides being developed. These nano-agrochemicals are now popular because they are more effective than conventional agrochemicals, making them both economically and environmentally viable [138].

As a result, it is safe to assume that this technology will be at the forefront of major markets, with more investment and innovation. Nano-agrochemicals, on the other hand, are still in their infancy and face obstacles in reaching farmers, with plausible causes being higher production costs, a lack of awareness among farmers, environmental and human effects, and so on. Novel agro-formulations with better benefits, such as organic-based nano-materials, are expected to revolutionize and improve agriculture to a greater extent around the world in the near future [137].

Farming contamination has been triggered by modern agricultural techniques. Due to modern-day agricultural by-products, this process has the potential to degrade ecosystems, land, and the environment. Agriculture is further harmed by the widespread use of chemical fertilizers, pesticides, and contaminated water for irrigation. As a result, the farm and food sector’s current situation are unsustainable. Nanotechnology has expanded the agricultural sector’s innovative and resourceful horizons by bringing practical applications to traditional agricultural methods and practices [139]. Traditional agricultural techniques have been revolutionized by the potential use of nanoscale agrochemicals, such as nano-fertilizers, nano-pesticides, nano-sensors, and nano-formulations in agriculture. The use of these nano-products in real-world scenarios, however, raises concerns regarding nanomaterial safety, exposure levels, and toxicological consequences for the environment and human health [140].

### 5.10. Nano Fertilizers

One of the potentially successful methods for significantly increasing worldwide agricultural productions, needed to fulfil the future demands of the growing population, is the development and use of new types of fertilizers employing creative nanotechnology. Indeed, a study of the current literature suggests that some manufactured nanomaterials can boost plant growth in specific concentration ranges and could be utilized as nano-fertilizers in agriculture to boost agricultural yields and/or reduce pollution. This article divides macronutrient nano-fertilizers, micronutrient nano-fertilizers, nutrient-loaded nano-fertilizers, and plant-growth-enhancing nanomaterials into four groups.

Macronutrient nano-fertilizers are made up of one or more macronutrient components (e.g., N, P, K, Mg, and Ca) and can, thus, provide these critical nutrients to plants. Large amounts of macronutrient fertilizers (mostly N and P fertilizers) are used to boost food, fiber, and other critical commodity production [141].

### 5.11. Micronutrient Nano-Fertilizers

These composite fertilizers usually include enough micronutrients to offer enough nutrition while posing little environmental hazards. However, in some soils with an alkaline pH, coarse texture, or low soil organic matter, plant availability of applied micro-nutrients may be poor, resulting in micro-nutrient insufficiency [134]. Even under these worst-case conditions, micronutrient nano-fertilizers may increase the bioavailability of these nutrients to plants. Nano-fertilizer production and implementation are still in their early phases, therefore, there are few, if any, particular studies or systematic studies on the effects and benefits of using micronutrient nano-fertilizers in the field [114].

### 5.12. Nano Pesticides

Nano pesticide formulations (such as emulsifiable concentrates, oil in water emulsions, microemulsions, nano-emulsions, and nano-dispersions) can be used in a variety of ways to boost the solubility of water-insoluble substances [142]. The emulsifiable nano-pesticide concentrate is made up of a pesticide, an organic solvent, an emulsifier, and a few other additions. Oil in water emulsion, a substitute for emulsifiable concentrate, is made by dissolving insecticide in nonionic and polymeric surfactants, as well as block polymers. Microemulsions are nano pesticide formulations with particle diameters 250 times smaller than regular pesticide particles [143].

Microemulsions provide a number of advantages, including improved solubility, reduced phytotoxicity, and improved thermodynamic stability [144]. Nano-emulsions are pesticide formulations based on nanotechnology that use a smaller amount of surfactants [145]. The particle size ranges from 20 to 200 μm. Although nano-emulsions are not thermodynamically stable, they are an excellent substitute for microemulsions [146]. Nano dispersion is a mixture of nano-crystals and liquid media that creates a larger surface area, allowing the poorly water-soluble nano-crystals to completely dissolve in water. Nanocrystals with a size of less than 50 nm dissolve more easily in water [147].

### 5.13. Nano Biosensors

Nano-biosensors are nano-sized sensors that have changed agriculture. These sensors are significant because they help to increase agricultural outputs and administer nano-based agrochemicals such as nano fertilizers and nano insecticides. Nano biosensors can detect physical and environmental elements in the plant’s environment, such as temperature, pH, humidity, soil parameters, moisture content, and the organic environment in the plant’s environment, such as plant-microbe interaction analysis, aflatoxins presence, and seed viability [148,149]. Work on biosensors began in 1962, and the fourth generation of biosensors has already been introduced as a result of different nano-based improvements. Nano-based formulations entail particle size reductions of up to a billionth of a meter, or 10-9. The first generation of biosensors created electrical signals as their output, while the present fourth generation of biosensors is rich in nano-based alteration and produces the best electrochemical response [150].

Nanotechnology-based biosensors can be divided into several categories. The electrochemical biosensors provide an electrochemical signal as a response. The creation of electrochemical signals is connected with the consumption of ions and electrons by the target element [150].

## 6. Applications of ZnO

Zinc oxide is harmless, and used to degrade the contaminated material of the environment [151]. Food and Drug Administration classified ZnO as a “generally regarded as safe (GRAS)” material that is also utilized as a food stabilizer. Zinc oxide (ZnO) nanoparticles are preferred over other metal oxide nanoparticles due to their wide range of uses, including gas sensors, biosensors, cosmetics, storage, solar cells, and medication administration [152].

### 6.1. Cancer Treatment Using ZnO-NPs

As a biomarker in similar ex vivo experiments, ZnO-NPs were demonstrated to have a great degree of cancer cell selectivity, surpassing therapeutic directories of certain regularly used chemotherapeutic drugs [126,153].

This indiscriminate activity frequently results in toxicity and devastating side effects in normal human tissues, all of which limit the chemotherapeutic drug’s maximum allowed dose [154]. The increased permeation and retention effect is a phenomenon in which the size of the NPs facilitates their entrance into the tumor cells. The utilization of the EPR effect in therapeutic techniques is now considered the “gold standard” in the development of novel anticancer drugs. The phenomenon of EPR explained as a changes in angiogenic regulators [133].

This localized imbalance permits nanoparticles of specific sizes to penetrate the tumor interstitial space with ease, but remain passively held, enhancing therapeutic potential. Biomarkers made of ZnO and the other metal oxide nanoparticles for cancer diagnosis, screening, and imaging. ZnO-NPs encapsulated with polymethyl methacrylate have been proven to be beneficial in the detection of low abundant biomarkers in recent research [107].

### 6.2. Applications in Biomedicine

Solids and powders of ZnO nano powders are available. Antifungal, anti-corrosive, antibacterial, and anti-corrosive capabilities are all present in these nanoparticles.

### 6.3. Antimicrobial Properties (Anti-Fungal and Anti-Bacterial)

Metal oxide (ZnO-NPs) powders were tested in culture conditions for the activity of antimicrobial against the bacteria and fungi. The larger surface area to volume ratio of these tiny particles accounts for their increased bioactivity. Against harmful microbes, ZnO nanoparticles are an excellent antimicrobial agent [36]. Basically, the antibacterial activity of these metal oxide particles could be attributed to the active oxygen species produced by them. Increased pathogenic strain outbreaks and infections, antibiotic resistance, the introduction of new mutations, the lack of an appropriate vaccination in developing countries, and hospital-associated illnesses are all global health threats to humans, especially in children [134]. The infections caused by *Shigella flexneri*, for example, result in 1.5 million fatalities each year as a result of contaminated food and drinks [155]. The vast range of applications of ZnO-NPs as an antibacterial agent resulted from research including, biologists, chemists, and medicine. One of the most important applications is in the food business, where it is used as an antibacterial agent against food-borne pathogens. Nanomaterials are attracting a lot of attention in the food industry due to their great reactivity, increased bio-availability and bio-activity, and unique surface properties [156]. The incorporation of NPs on the food surface to limit bacterial development is one of the key benefits of employing NPs in food nanotechnology [157]. The ZnO-NPs antibacterial activity contains the interaction between the zinc oxide and the surface of the cell that can change the permeability of the cell membrane; then, these nanoparticles inter in the bacterial cell. In bacterial cells, these nanoparticles cause oxidative stress, which can inhibit the growth of cells and can also cause the death of that cell [158]. This activity of nanoparticles shows that these ZnO-NPs are also used in the food industry to clean the equipment and to protect food from bacterial disease [159].

### 6.4. The Function of ZnO-NPs in the Agriculture

Agriculture is the backbone of third-world economies. It is facing a number of challenges, such as climate change, urbanization, sustainable resource use, and environmental issues, such as runoff, pesticide accumulation, fertilizer, and the population of the world that is increasing gradually and is expected to increase at a large scale in the future. As a result, in order to make agriculture more sustainable, we must implement efficient practices [109]. Nanotechnology techniques are altering agriculture and food production in many ways. These techniques have the potential to change the forming techniques. It is effective in controlling the damage from pesticides and fertilizers. That is why it increases food production and improves the growth of all crops. This is the less expensive technique to reduce the damage [34,108,160].

The creation of nano-sensors aid in estimating the amount of the farm inputs like fertilizers and pesticides that are necessary. The moisture content of the soil and the nutrients that are present in the soil can also be detected by these nanosensors [161,162]. Nano fertilizers are quickly absorbed by plants. Slow-release nano encapsulated fertilizers can reduce fertilizer use while also reducing pollution. ZnO-NPs have the potential to enhance food crop productivity and growth. Different quantities of zinc oxide nanoparticles were applied to peanut seeds. Seed germination, seedling vigor, and plant growth were all improved with a ZnO nanoscale treatment at 1000 ppm concentration, and these zinc oxide nanoparticles were also beneficial in enhancing stem and root growth in the peanuts plants [41].

Nano fertilizers are used to provide nutrients to the plants and can also restore the soil. These are more effective than common fertilizers that are used in plants. Without any adverse effects of chemical fertilizers, they can restore the soil’s organic conditions. Nano fertilizers have the advantage of being able to be applied in extremely small doses. Ordinary fertilizers would require 150 kg for an adult tree; however, organic fertilizers only require 40–50 kg. Nano powders have also been utilized successfully as fertilizers and herbicides. The yield of wheat plants developed from metal nanoparticle-treated seeds rose by 20–25% on average [163]. Nano fertilizers have the advantage of being able to be applied in extremely small doses. Ordinary fertilizers would require 150 kg for an adult tree; however, organic fertilizers only require 40–50 kg. Nano powders can be utilized as fertilizers and pesticides with success [164]. ZnO nanoparticles are employed in nano fertilizers, and colloidal solutions of ZnO NPs are used in agriculture. Crops that are treated with these nanoparticles grow faster and produce more. The output of staple food crops is substantially lower as food demand rises day by day. Metal NPs for sustainable agriculture are therefore urgently needed [164].

### 6.5. Use in Water Treatment

Nanoparticles are projected to play a critical part in water filtration. The environmental destiny and the toxicity of the substance are important thoughts in water purification material selection and design [165,166]. Although nanotechnology is undoubtedly high to existing water treatment techniques, our understanding of the environmental destiny and toxicity of nanoparticles is still in its start [167].

Most of the present issues with water quality are remedied or considerably reduced by using non-absorbent nano-catalysts, bio-active nanoparticles, nanostructured catalytic membranes, and other nanoscale science and engineering advances. Metal nutrients, cyanide organics, algae, bacteria, parasites, viruses, anti-biotics, and biological agents are utilized for terrorism. The development of innovative desalination methods is one of the common intriguing and promising areas of the research [168]. Due to its simplicity, low cost, ease of parameter control, and great effectiveness in degrading organic and inorganic compounds in aqueous systems, photocatalytic methods have been investigated [166].

### 6.6. Effects of ZnO-NPs on the Plant Growth

**Figure 6:**
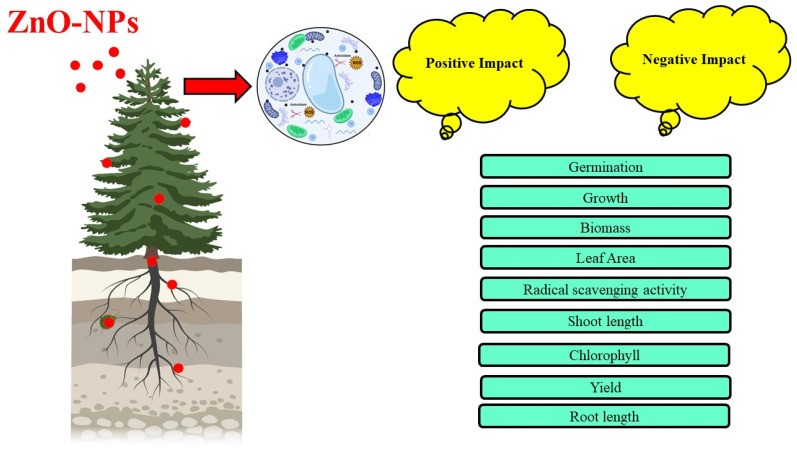
Effect of Zinc Oxide Nanoparticles on plant growth.

The increasing production and applications of engineered NPs have generated an emerging area of research that focuses on their environmental and ecological impacts [21]. Numerous studies have shown metal-based NPs may result in accumulation of themselves and/or the component metal in edible plants [51], either reduce or improve crops’ yield and productivity [169], and sometimes negatively impact soil microbial communities and activity [108]. Due to their high specific surface area and complexing capability, NPs may adsorb pollutants, subsequently changing the transport, bioavailability and toxicity of both the NPs and the pollutants. NPs are compounds with diameters ranging from 1 to 100 nm that have become extensively used in recent years. Organisms consider ZnO-NPs to be a “bio safe substance.” At various developmental stages, ZnO-NPs show the ability to germinate the seed and to stimulate plant growth (Figure 6), and it also decreases disease infection, due to their antibacterial action. ZnO-NPs show positive and negative effects on the growth of plants and many metabolic activities at various developmental stages. The characteristics of ZnO-NPs, influence their uptake, transport, and accumulation by plants [119,122].

NPs of various metal oxides can help plants grow and produce more, but research into the toxicological effects of NPs is increasing all the time. Studies are undertaken to determine the effects of ZnO-NPs on the plants [170]. In the presence of ZnO-NPs, ryegrass biomass dramatically decreased, root tips shrank, and root epidermal cortical cells became extensively vacuolated, according to toxicological tests. Individual ZnO-NPs found in the apoplast and protoplast of root endodermis and stele in wheat treated with ZnO-NPs were translocated [26,171].

### 6.7. ZnO-NPs Have Negative or Toxic Effects

Due to the increasing use of NPs and their release in the environment, it is necessary to determine the toxicity of nanoparticles. Vicario-Pares et al. [172] conducted a toxicity study of three metal oxide NPs, namely, CuO NPs (copper oxide nanoparticles), ZnO NPs, and TiO_2_ NPs against zebra fish embryo. ZnO-NPs were found to be less toxic than the ionic form of Zn, which exerts the highest toxicity. Further, ZnO-NPs were found to exhibit a higher antibacterial activity against *Staphylococcus epidermidis* and *Enterobacter aerogenes*. Results of the toxicity study show that ZnO-NPs at a concentration of 10 mg/mL did not show any significant effect on survival and malformation in the zebra fish embryo [173]. In spite of the fact that ZnO-NPs are important commercially and are found in a variety of products, there is an increasing public interest in learning more about their toxic and environmental impacts. Research on ZnO nano-particles showed that it may pose health and eco concerns [174].

## 7. Application of ZnO Reduce the Heavy Metal Stress

Most heavy metals are produced by pollution and their presence causes many ecological, evolutionary, and nutritional problems [175,176,177,178]. Many risks are created due to heavy metal contamination, like soil pollution, as well as security of food and its quality [86,179,180,181,182]. Heavy metals cause many harmful effects for living things, including plants [92,183,184]. They decrease the growth and development of plants even at low concentrations of heavy metals with respect to other metals [185,186,187]. Excess amounts of other metals or elements do not damage the tissues/cells of the plant, and their accumulation can even increase the growth of the plant [188,189,190,191]. Metals which are lethal or harmful for plants include Pb, Cd, Co, Fe, Hg, Pt, Ni, Cr, Cu, and Zn [44,192]. Agricultural soil is befouled by many heavy metals, which is a major issue. Many anthropogenic activities, such as urbanization, smelting, sludge, military operations, mining, dumping, and excess amounts of pesticide and insecticide applications, affect soil and the effectiveness of the plant [68,193].

Recently, there has been a flow of interest in studying the effect of nanoparticles on the reduction of heavy metal toxicity. Heavy metals (Cd, Pb, Ag, CU, and Zn), nutrients, cyanide, and the other organics are detected and removed using a variety of nanomaterials like that nano-particles, nanomembranes, and nano powder [164]. Phytoremediation researchers are testing whether metal nanoparticle amendment can promote hyperaccumulation without damaging plant biomass, thanks to the use of nano-technology in water the purification and supply systems of water [194]. The current study examines the impact of Phyto molecule-loaded ZnO-NPs in Cd and Pb hyperaccumulation in *Leucaena leucocephala*. ZnO-NPs have unique optical and electrical properties that can be employed in a range of applications, including coatings to remove harmful chemical and biological contaminants, such as heavy metals [108]. After 3 days of remediation, Mohsenzadeh and Rad [195] measured the efficiency of plant-derived Zn nanoparticles and found a lower level of Pb and Cd heavy metals in the polluted water. Ma et al. [196] summarized the effect of metallic nano-particles. It also showed that some adverse effects on plant growth affect the metabolic activities of some higher plants. It has a negative effect on some physiological functions. It inhibits the root growth, decreases the chlorophyll content, and the delays the development of plant. The effect of ZnO-NPs with different sources on the growth and eco-physiology of the plants are presented in Table 3.

## 8. Conclusions

Zn is a micronutrient, and standard zinc fertilizers (among others) have a low bioavailability problem due to the element’s fixation to compounds in the soil that are insoluble. Enlightening our understanding of different forms of Zn, as well as uptake and the assimilation of Zn by higher plants, could be the first step toward more widespread use of ZnO-NPs in agriculture for plant nutrition and protection. When discussing nano fertilizers and other goods, we must not overlook their toxicity, which is one of the most significant barriers to their adoption. Plant systems capable of limiting and reducing the harmful effects of Zn-NPs should be the focus of study in this area. Furthermore, during toxicity tests, we should avoid using excessively high doses of Zn-NPs. Plants require modest levels of Zn (and other minerals) to grow and develop properly. In the recent decade, synthesis of the NPs using an environmentally benign manner has been a focus of study. For the production of shape and size-regulated nanoparticles, green sources operate as both a stabilizing and a reducing agent. Extension of laboratory-based studies to industrial scale, clarification of phytochemicals involved in NPs synthesis using the bioinformatics techniques, and derivation of the exact mechanism involved in pathogenic bacteria inhibition are all future prospects for plant-mediated NPs synthesis. Plant-based nanoparticles offer a wide range of applications in food and pharmaceuticals and have, thus, become a prominent study topic.

## Figures and Tables

**Table 1:** Effect of Zn application on growth and eco-physiology of the various plant species under the treatment of various heavy metals in the soil.

Plant Species	Metal Type	Culture	Metal Duration (Days)	Comments	References
Yellow Lupine	Cd	Soil	Full maturity	Zn application enhanced plant yield under metal stress	[105]
*Brassica napus*	Cd	Hydroponic	14	Depending upon the different cultivars, the shoots Cd was decreased	[106]
*Triticum aestivum*	Cd	Soil	125	Application of Zn enhanced eco-physiology of the plant	[6]
*Oryza sativa*	Cr	Soil	70	Application of Zn enhanced growth and decreased Cr contents	[42]
*Triticum aestivum*	Cr	Soil	120	Zn application decreased oxidative damaged in the membrane bounded organelles	[107]
*Oryza sativa*	As	Soil	50	ZnO regulated various transcriptional pathways participated in oxidative stress tolerance	[108]
*Glycine max*	As	Soil	Maturity	As stress inhibited growth and photosynthesis, but regulated by the application of ZnO	[109]
*Glycine max*	As	Soil	60	ZnO application decreased As concentration in the roots and shoots of the plants	[110]
*Morus alba*	Pb	Soil	90	Zn improved gas exchange capacity, increasing growth and biomass, and improved redox imbalance in the plants	[37]

**Table 2:** Methods for the synthesis of ZnO-NPs.

Methods	Process	Advantages	Disadvantages	References
Chemical synthesis	Spray pyrolysis, thermal breakdown, molecular beam epitaxy, chemical vapor deposition.	It is the most significant proces, and it is performed with a variety of precursors and under a variety of variables. The size and geometries of NPs are morphologically changed	Hazardous compounds adsorbed on the surface, which could have negative consequences.	[128]
Vapor transport synthesis	Zinc and oxygen vapors react with each other	It is the most prevalent method and growth temperature is relatively moderate.	Imbalance vapor pressure ratio may affect the ZnO nanostructure.	[129]
Hydrothermal synthesis	Low temperature process	The use of simple equipment, catalyst-free growth, low cost, homogeneous production, Eco friendliness, and being less toxic.	May require high temperature to initiate.	[130]
Green synthesis	plant components such as the leaf, and other parts	This is a very environment friendly, low-cost method that does not require the use of intermediate base groups.		[131]
Bacterial based synthesis	Green synthesis	Increased photocatalytic activity when compared to other substances, which destroys organic waste and can, thus, be utilized as a bioremediation method.	Time-consuming microbe screening, careful monitoring to avoid contamination.	[132]

**Table 3:** Effect of ZnO-NPs on plant species.

Plant Species	Application of Nanoparticles	Effects	References
*Zea mays*	Foliar spray	Grain yield increased and zinc content of grain also increased	[197]
*Oryza sativa*	Plant agar	Growth increased	[198]
*Glycin max*	Paper (petri dishes)	Seedling growth inhibited	[199]
*Phaseolous vulgaris*	Foliar spray	All the growth parameters prompted and increased the content of guar gun	[132]
*Solanum lycopersicum*	substrate	It reduced the chlorophyll and the activity of antioxidants increased	[115]
*Pisum sativum*	substrate	sucrose, carotenoids and chlorophyll content increased	[126]
*Arabidopsis thaliana*	Plant agar	Germination and growth of seedling inhibited	[130]
*Vigna radiate*	Plant agar	Seedling growth promoted at <20 mg/L concentration	[200]
*Arachis hypogea*	Foliar spray	Promote early flowering, increase the chlorophyll content, better sapling viability, germination also promoted	[41]

## Data Availability

Not applicable.
